# Active Peptide AR-9 From *Eupolyphaga sinensis* Reduces Blood Lipid and Hepatic Lipid Accumulation by Restoring Gut Flora and Its Metabolites in a High Fat Diet–Induced Hyperlipidemia Rat

**DOI:** 10.3389/fphar.2022.918505

**Published:** 2022-09-13

**Authors:** Hong Wang, Pingping Dong, Xin Liu, Zhen Zhang, Huajian Li, Yanan Li, Jiayu Zhang, Long Dai, Shaoping Wang

**Affiliations:** ^1^ School of Pharmacy, Binzhou Medical University, Yantai, China; ^2^ School of Pharmacy, Shandong University of Traditional Chinese Medicine, Jinan, China; ^3^ State Key Laboratory for Quality Research of Chinese Medicines, Macau University of Science and Technology, Avenida Wai Long, Macao SAR, China

**Keywords:** hyperlipidemia, *Eupolyphaga sinensis*, AR-9, metabolomics, gut flora, Spearman analysis

## Abstract

The dysbiosis of gut flora and its metabolites plays important roles in the progression of hyperlipidemia (HL), and some bioactive peptides are available for HL treatment. In this study, we aimed to isolate an active peptide (AR-9) from active peptides of *E. sinensis* (APE) and determine whether AR-9 could improve many symptoms of a HL rat induced by a high-fat diet (HFD) by modulating gut flora and its metabolites. Above all, AR-9 was derived from APE using ion-exchange chromatography, and its structure was deconstructed by Fourier transform infrared spectrometer (FT-IR), circular dichroism (CD) spectroscopy, and UHPLC-Q-Exactive-Orbitrap MS. Then, an HFD-induced HL model in SD rats was established and used to clarify the regulatory effects of AR-9 (dose of 3 mg/kg) on HL. Normal diet–fed rats were taken as the control. The plasma samples and liver were harvested for biochemical and histopathological examinations. 16S rRNA gene sequencing and untargeted metabolomics were sequenced to assess changes in gut flora and its metabolites from rat fecal samples. Finally, Spearman’s correlation analysis was used to assess the relationship between lipid-related factors, gut flora, and its metabolites so as to evaluate the mechanism of AR-9 against HL. The results of the separation experiments showed that the amino acid sequence of AR-9 was AVFPSIVGR, which was a fragment of the actin protein from *Blattaria* insects. Moreover, HFD rats developed exaltation of index factors, liver lipid accumulation, and simple fibrosis for 8 weeks, and the profiles of gut flora and its metabolites were significantly altered. After treatment, AR-9 decreased the levels of lipid factors in plasma and the extent of liver damage. 16S rRNA gene sequencing results indicated that AR-9 significantly increased the relative abundance of beneficial bacteria *Bacteroidetes* and reduced the relative abundance of the obesity-associated bacteria *Firmicutes*. Furthermore, AR-9 changed gut microbiota composition and increased the relative abundance of beneficial bacteria: *Lactobacillus*, *Clostridium*, *Dehalobacterium*, and *Candidatus arthromitus*. Fecal metabolomics showed that the pathway regulated by AR-9 was “arginine biosynthesis”, in which the contents were citrulline and ornithine. Spearman’s correlation analysis revealed that two metabolites (ornithine and citrulline) showed significantly negative correlations with obesity-related parameters and positive correlations with the gut genera (*Clostridium*) enriched by AR-9. Overall, our results suggested interactions between gut microbial shifts and fecal amino acid/lipid metabolism and revealed the mechanisms underlying the anti-HL effect of AR-9. The abovementioned results not only reveal the initial anti-HL mechanism of AR-9 but also provide a theoretical basis for the continued development of AR-9.

## Introduction

Hyperlipidemia, which is accompanied by an increase in triglycerides (TGs), total cholesterol (TC), and low-density lipoprotein cholesterol (LDL-C), is a common clinical metabolic disease (Huang et al., 2019). In addition, with the change in people’s diet structure, it has become one of the major risks for many diseases, including hypertension, atherosclerosis, diabetes, vascular dementia, and coronary heart disease (Yao et al., 2020), and its incidence has increased significantly in all age groups (Owens et al., 2014).

Gut flora has been found to play an important role in regulating lipid metabolism *via* the changes in their composition and metabolites (Nicholson et al., 2012). In numerous previous studies on HL, the relative abundances of *Firmicutes* and *Bacteroidetes*, which are the most two abundant phyla of gut flora, were significantly altered. In the intestinal flora of patients with HL, the relative abundances of *Firmicutes* were significantly increased, while the content of *Bacteroidetes* was significantly decreased. At the genus level, the levels of beneficial bacteria, consisting of *Lactobacillus*, *Bacteroides,* and *Clostridium*, were decreased, while the levels of harmful bacteria, such as *Bacillus*, *Coccus* and *Streptococcus*, were increased (Indiani et al., 2018). Furthermore, gut flora is widely regarded as the largest endocrine organ of the human body, which can generate a variety of bioactive metabolites, such as amino acids, bile acids, lipids, phospholipids, and short-chain fatty acids (Nicholson et al., 2012). Among them, short-chain fatty acids and amino acids, which were generated by fermenting dietary fiber and protein by special bacteria (*Lactobacillus*, *Bifidobacterium,* and *Clostridium*), can inhibit lipid production and accelerate lipid oxidation by mediating signaling pathways to achieve regulation of lipid factors (Rom et al., 2020). Hence, the composition of gut flora and changes in its metabolites, which are revealed using appropriate technologies, are of great significance for the diagnosis of HL and the discovery of the mechanism after drug treatment. However, it is a challenge to fully demonstrate the composition of gut flora using traditional microbiological techniques. Also, the levels of metabolites from gut flora are also difficult to be detected by conventional analytical methods with factors of low response and impurity interference. Nowadays, 16S rRNA sequencing technology has been widely used in the identification of intestinal flora (Alanee et al., 2019). Moreover, metabolomics based on ultra-high performance liquid chromatography-high resolution mass spectrometry (UHPLC-HRMS) has also been used to excavate the types and content changes of metabolites (Li et al., 2017).

Tubiechong (*Eupolyphaga sinensis* Walker, *E. sinensis*) used in traditional Chinese medicine (TCM) was first recorded in Shennong’s Herbal Classic written during the Han dynasty (Liu et al., 2019). Many reports have indicated that *E. sinensis* shows multiple pharmacological effects, such as reducing blood stasis, anticoagulant, antitumor, anti-mutation, and hypoxia tolerance. The fact that protein is the main component of *E. sinensis* has been found in previous experiments (Liu et al., 2019; Zhang et al., 2019). It is well-known that a protein cannot be directly absorbed by the body because of its high molecular weight and complex structure. In contrast, the peptides are much smaller than large proteins and they can easily fulfill the need for mid-size therapeutics with *in vivo* absorption efficiency (Shen et al., 2017). Therefore, bioactive peptides should show stronger biological activities than the precursor protein.

Based on the hypolipidemic effect and high protein reserves of *E. sinensis*, in this study, the APE was first obtained by biomimetic enzymatic hydrolysis consisting of pepsin and trypsin. Then, a range of separation techniques were used to excavate and analyze AR-9 in APE. Furthermore, the structure and source of AR-9 were simultaneously resolved based on chemical composition analysis methods and related databases. At the same time, a HFD-induced-rat model was used to evaluate the different activities of AR-9 and APE on anti-HL. Moreover, the possible mechanism of AR-9 against HL will be initially predicted by 16S rRNA sequencing technology and the untargeted metabolite profiling combined with Spearman’s analysis. Therefore, the present study aimed to assess the effects of AR-9 on HL induced by a high-fat diet and its influence on the gut microbiota and its metabolites in HL rats.

## Material and Methods

### Materials

*E. sinensis* was purchased from Hebei Hongsen Pharmaceutical Co., Ltd (Hebei, China). The specimens were preserved at the chemical laboratory of Binzhou Medical University. Pepsin (2,500 U/mg) and trypsin (5,000 U/mg) were all obtained from Shanghai Sinopharm Reagent Group Co., Ltd (Shanghai, China). The kits of total cholesterol (TC), triglyceride (TG), high-density lipoprotein cholesterol (HDL-C), and low-density lipoprotein cholesterol (LDL-C) were purchased from Nanjing Jiancheng Bioengineering Institute (Nanjing, China).

### Isolation and Purification of AR-9

*E. sinensis* after pulverization was added into deionized water to the concentration of 10% (weight/volume). After being denatured for 10 min at 90°C and cooled to 40°C, the pH of the sample was adjusted to 2.0 with 1.0 M HCl. The solution was then supplemented with pepsin (1.0% of the drug) and subjected to enzymatic hydrolysis for 1.0 h. Thereafter, the pH of the primary solution was adjusted to 8.0 with 1.0 M NaOH before the addition of trypsin (1.0% of drug), followed by incubation for 3.0 h with stirring. Finally, the enzymatic solution was soaked in boiling water for 15 min to inactivate the enzymes. The mixture was then centrifuged at 5,000 rpm for 10 min.

The resulting supernatants were separated into different constituents using an ultrafiltration cartridge of a 3,000 Da nominal molecular weight cut-offs (NMWC) membrane (Shanghai Mosu Scientific Equipment Co., Ltd). For salt removal, the abovementioned solution was passed through 500-Da dialysis membranes and lyophilized. The lyophilized sample was redissolved with deionized water and further isolated using a DEAE-Sepharose Fast Flow anion-exchange column (2.6 × 40 cm). The column was equilibrated with 0.03 mM Tris-HCl buffer (pH 8.0). The sample was added to the protein chromatography system (AKTA explorer 10.0) and eluted with a stepwise gradient of NaCl aqueous solutions (from 0.1 to 2 M) prepared in Tris-HCl buffer. The fractions were combined according to the number of elution peaks to gather them. After dialyzing, freeze-drying, and concentrating, the fractions which include AR-9 were obtained (Li et al., 2018; Singh et al., 2020).

The collected components were analyzed by reversed phase-high-performance liquid chromatography (RP-HPLC) to obtain the highest purity compound of AR-9. The separation was achieved on Waters symmetry C18 column (250 × 4.6 mm, 5 µm). The mobile phases consisted of (A) water containing 0.1% trifluoroacetic acid and (B) acetonitrile (ACE) containing 0.1% trifluoroacetic acid (Mant et al., 2016). The gradient of the mobile phases was set as follows: 0–20.0 min, 10–55% B; 20.0–25.0 min, 55% B; 25.0–26.0 min, 55–10% B; and 26.0–30.0 min, 10% B. The injection volume was 10 µl, and the wavelength was 220 nm.

### Structural Elucidation of AR-9

As we know, CD spectroscopy of protein is highly sensitive toward the secondary structure and the same applies for peptides. Therefore, the secondary structure of AR-9 was analyzed by CD spectroscopy using the reported method (Liu et al., 2020). Moreover, the types of functional groups contained in AR-9 could be detected for FT-IR (Hackshaw et al., 2020). The AR-9 mixed with potassium bromide was flaked and then measured using an FT-IR spectrometer (Shimadzu IRTracer-100, JPN) in the wavelength range of 4,000–400 cm^−1^ at 30°C.

Finally, the complete amino acid sequence of AR-9 was determined by an Easy nLC1200-Q-Eaxtive plus mass spectrometer (Thermo Fisher Scientific, MA, United States) which contained a binary pump, an autosampler, and a column oven. The bioZen^TM^ peptide XB C18 column (150 × 0.075 mm, 2.6 µm) was used as a separation medium, mobile phase A was deionized water (0.1% formic acid, FA), ACE with 0.1% FA was set as mobile phase B. Seven microliter AR-9 (10 mg dissolved by 100 ml 0.1% FA) was injected into the analysis platform and separated using a linear gradient elution: 0–3 min, 2–6% B; 3–42 min, 6–20% B; 42–47 min, 20–35% B; 47–48 min, 35–100% B; 48–60 min, 100% B. The flow rate was 300 nL/min.

The ESI-MS/MS information of AR-9 was collected using a Q-Exactive Orbitrap mass spectrometer (Thermo Fisher Scientific, MA, United States) with a data-dependent scan mode. The mass range of the MS scan was set to *m/z* 350–1,800 in the positive mode with a resolution of 70,000, and the precursor ion of AR-9 (Charge state ≥+1) was fragmented with the high-energy collision of 28.0 NEC. The temperature of the capillary was 275°C, and the spray voltage was set as 2,200 V. The product ions were measured on an orbit with a resolution of 17,500 (AGC 1e5). For dynamic exclusion, the specific parameters were as follows: a repeat duration of 25 s and exclusion duration of 25 s.

### Establishment of an HL Rat Model and Treatments

A total of 30 male Sprague–Dawley (SD) rats (SPF level, weighing 180–200 g) were purchased from Jinan Pengyue Experimental Animal Breeding Co., Ltd (Shandong, China, SYXK (RU)2019-0003). Before experimental interventions, all animals must be maintained under standard animal room conditions (temperature 24 ± 2°C, humidity 55–60%, 12/12 h light/dark cycles) with standard feed and water *ad libitum* for 1 week. Afterward, all rats were randomly divided into the control group (Con, six rats) and the HL group (24 rats) according to body weight. The rats in the control group were fed normal rodent chow (Pengyue, Shandong, China), and those in the HL group were fed with a HFD, containing a standard chow diet (65%), sucrose (20%), lard (15%), cholesterol (5%), sodium cholate (5%), and 5% yolk powder (Huafukang, Beijing, China) for 8 weeks (Zheng et al., 2020). Then, the rats in the HL group were again allocated into the four following groups: the model group (Mod, *n* = 6), simvastatin group (Sim, *n* = 6) at the dose of 5 mg/kg/d (drug weight/body weight/day), APE group (APE, *n* = 6) at the dose of 25 mg/kg/d, and AR-9 group (AR-9, *n* = 6) at the dose of 3 mg/kg/d. This dose was determined based on the percentage of AR-9 in APE (12% of APE by area normalization method, Supplementary Table S1). Except for the Con group, other rats were still fed a HFD during 3 weeks of treatment.

### Collections and Preparation of Biological Samples

After the final gavage, all rats were starved for 12 h, receiving only deionized water. Fresh feces from all rats were collected and stored at −80°C refrigerators for fecal metabolomics and intestinal microbial analysis. The rats of five groups were killed in parallel using 10% pentobarbital sodium by intraperitoneal injection. The blood samples were collected from the abdominal aorta and were centrifuged (3,500 rpm for 15 min, 4°C) to obtain plasma for biochemical analysis (TC, TG, LDL-C, and HDL-C). The tissues of the liver were fixed in 4% paraformaldehyde fixation (PFA) (Servicebio, Wuhan, China) for subsequent analysis.

### Analysis of Plasma Cytokines and Liver Histopathological Examination

The levels of TG, TC, LDL-C, and HDL-C in plasma samples from all rats were measured using a microplate reader (SpectraMax iD5, MD, United States). The hepatic tissues fixed in 4% PFA were dehydrated and embedded in paraffin, cross-sectioned into 4-µm-thick slices, and stained with hematoxylin–eosin (H&E). The sections of the remaining liver tissues were cleaned with PBS and cultured with 60% isopropanol for 5 min and then dyed in 0.5% Oil Red O staining liquid (Sigma, MO, United States) for 20 min. After being cleaned by PBS, all sections were then stained with hematoxylin stain (Solarbio Science and Technology, Beijing, China) for 2 min. The abovementioned indicators were all used to evaluate the anti-HL function of AR-9.

### Untargeted Metabolomic Analysis of Gut Flora Metabolites

#### Sample Preparation

The fecal pellets stored at −80°C were freeze-dried and crushed. 100 mg powders were ultrasonically extracted with 500 µl cold water and 500 µl cold ethanol for 30 min in succession. Then, the two extracts were combined and centrifuged (10,000 rpm for 15 min, 4°C); the resulting supernatant was collected and evaporated by nitrogen at 4°C (Yu et al., 2017). These residues were redissolved using 100 µl of 50% methanol and centrifuged at 4°C for 15 min at 15,000 rpm to obtain the supernatant for further analysis. In addition, a 10 µl solution from each fecal sample was mixed and marked as quality control (QC) samples. The stability of the instrument needed to be calibrated using QC samples after every five fecal samples.

#### Data Acquisition for Fecal Untargeted Metabolomics

Chromatographic separation was performed on a Dionex Ultimate 3000 UHPLC system equipped with a WPS-3000 TRS autosampler, a TCC-3000RS column oven, and an HPG-3400RS binary pump (Dionex Softron GmbH-Part of Thermo Fisher Scientific, Germany). An ACQUITY UPLC BEH HILIC column (150 mm × 2.1 mm, 1.7 µm) was operated at 40°C. The mobile phase comprised (A) 0.1% FA in water and (B) ACE with a flow rate of 0.3 ml/min. The elution parameters were 0.00–5.00 min, 5% A; 5.00–6.00 min, 5–20% A; 6.00–10.00 min, 20–25%; 10.00–13.00 min, 25–40%; 13.00–16.00 min, 40%; 16.00–16.10 min, 40–5%; 16.10–20.00 min, 5% A.

The ion spray voltages were set at 3.5 kV in the positive ion mode and 3.0 kV in the negative ion mode. The auxiliary gas flow was 10 arb, sheath gas flow was 30 arb, the mass range of the MS scan was *m/z* 70–1,050, auxiliary gas heater temperature was 350°C, and capillary temperature was 320°C.

#### Multivariate Analysis of LC-MS/MS Data

The LC-MS/MS data were processed with Compound Discoverer 3.0 software (Thermo Fisher Scientific, MA, United States) for noise cancellation, baseline correction, and normalization to obtain reliable data sets with some information, including *m/z*, peak intensity, and retention time.

Afterward, the processed data sets were added to SIMCA-P 14.0 software (Umetrics, Sweden) for performing the principal component analysis (PCA) and orthogonal to partial least squares-discriminate analysis (OPLS-DA). Among them, PCA was applied to discriminate the interesting separation trend of all groups. OPLS-DA was used to characterize metabolic perturbation of HL. In addition, S-plots were used to screen the differential metabolites of HL treated by AR-9 combined with other judgment methods, such as variable importance in projection (VIP) (generated in the OPLS-DA mode) and *p*-value (formed from relative intensity). The structures, molecular weights, and codes of differential metabolites were assigned according to the human metabolome database (https://hmdb.ca/).

### Intestinal Microbiota Analysis by 16S rRNA Gene–Based Amplicon Sequencing

#### Fecal DNA Extraction and PCR Amplification

Bacterial DNA was isolated from fecal samples using the cetyltrimethylammonium bromide (CTAB) method (He et al., 2018). The DNA concentration from all samples was detected by 1% agarose gels. Then, DNA was diluted to 1 ng/μl using sterile water. The extracted DNA from each sample was used as the template to amplify the V3–V4 region of 16S rRNA genes using PCR (Zhang et al., 2016). All PCR reactions were performed in 30-µl reactions with 15 µl of Phusion® High-Fidelity PCR Master Mix with GC Buffer (New England Biolabs), 0.2 µM of forward and reverse primers, and approximately 10 ng of template DNA. The purity of PCR products was examined using electrophoresis (2% agarose gels).

#### 16S rRNA Gene–Based Amplicon Sequencing and Bioinformatic Analysis

The sequencing libraries were generated using the TruSeq® DNA PCR-Free Sample Preparation Kit (Illumina, United States) according to the manufacturer’s recommendations, and index codes were added. The library quality was assessed on the Qubit @ 2.0 Fluorometer (Thermo Scientific). At last, the library was sequenced on an Illumina NovaSeq platform.

Appropriate methods including ANOVA and LEfSe were used to identify the bacteria with different abundance among samples and groups (Love et al., 2014; Mandal et al., 2015). Alpha diversity and beta diversity were calculated using the core-diversity plugin within QIIME2 (Hall et al., 2018). Among them, the alpha diversity indices, such as observed operational taxonomic units (OTUs), Chao1 richness estimator, Shannon’s diversity index, and Faith’s Phylogenetic Distance, were calculated to estimate the microbial diversity within an individual sample. The observed OTUs and Chao1 indices reflect the abundance of species and, in addition, the Shannon and Simpson indices were applied to measure the abundance and diversity of the species contained in the samples (Yang et al., 2019). The beta diversities were manifested as PCoA and NMDS analysis. LEfSe analysis was used to compare species with significant differences among groups.

### Statistical Analysis

All results were analyzed using statistical product and service solutions (SPSS) 22.0 software and were presented as Mean ± SD. One-way ANOVA and Student’s *t*-test were used to assess statistical significance for multiple comparisons among the different groups. *p* < 0.05 and *p* < 0.01 were defined as statistically significant. The statistical analyses and figures were performed using GraphPad Prism 8.0 software.

The Spearman correlation analysis was performed to determine the potential correlations of the intestinal microbial, differential metabolites, and biochemical indicators. The correlations of the abovementioned three variables were visually displayed using heat maps, separately. Finally, the intestinal microbes and biomarkers that reflect the anti-HL function of AR-9 were extracted.

## Result

### Isolation, Purification, and Structural Elucidation of AR-9

**FIGURE 1:**
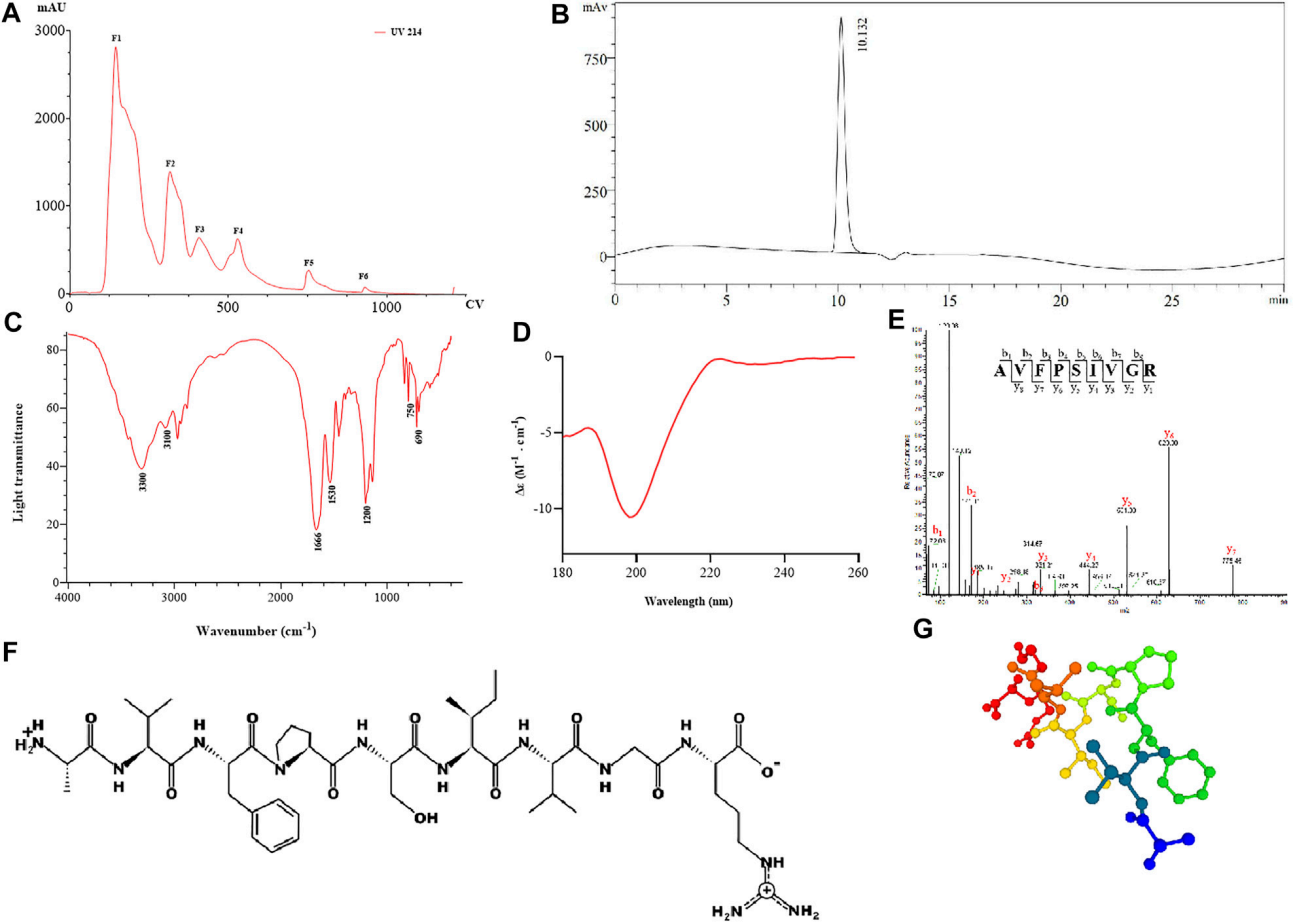
Results of separation and structural analysis of AR-9. **(A)** Anion-exchange chromatography elution curve, **(B)** RP-HPLC of F1-F6, **(C)** IR spectrum of AR-9, **(D)** CD spectrum of AR-9, **(E)** corresponding sequence was determined from this spectrum as AVFPSIVGR, **(F)** and **(G)** molecular structure and conformational prediction of AR-9.

The result of APE separated by anion-exchange chromatography is shown in Figure 1A, and six fractions were obtained and named F1–6, respectively. We then collected these fractions for peptide sequencing and found that AR-9 was the most abundant in relative abundance in F5 (Supplementary Table S2). The result of RP-HPLC indicated that the purity of AR-9 from F5 was 96.5% according to the area normalization method (Figure 1B).

FT-IR spectroscopy has been confirmed to be a valuable tool for providing information on protein secondary structural changes. Under normal conditions, a peptide can generate several infrared-active amide vibrational modes (Tatulian 2019): amide A (−3,300 cm^−1^), amide B (3,100–3,050 cm^−1^), amide I (1,700–1,600 cm^−1^), and amide II (1,570–1,540 cm^−1^). As shown in Figure 1C, the FT-IR spectrum of AR-9 exhibited a spectral band at 1,666 cm^−1^, which prompted the presence of α_Ⅱ_-helical structure in AR-9; the bands around 3,300 cm^−1^ and 3,100 cm^−1^ illustrated that they were generated by Fermi resonances between amide II and NH stretching vibrations. The amide I band mostly consists of C=O stretching vibrations and C-N groups, while the amide II band not only consists primarily of N–H bending but also C-N and C-C stretching vibrations (Tiernan et al., 2020). Moreover, broad absorption peaks at 750 cm^−1^, 690 cm^−1^, and 1,200 cm^−1^ were detected, indicating that AR-9 might comprise a benzene ring structure.

For CD analysis, almost all disordered proteins can be shown to have remarkably similar characteristics with a negative peak in the region of around 190–200 nm (Lopes et al., 2014). As shown in Figure 1D, the CD spectrum of AR-9 exhibited a strong negative band around 198 nm and a positive band around 223 nm. The analysis by the CDNN software calculates that the α-Helix accounts for 9.8%, the β-turn for 22.4%, the random coil for 32.8%, and the β-sheet for 34.9%.

LC-MS/MS is an available tool for the identification, quantification, and analysis of peptides. In the large majority of cases, the identification of peptide sequences has been enabled from collision-induced dissociation (CID) MS^2^ spectra using database search tools (Guthals et al., 2012). The resulting fragments associated with several b and y ions correlated to the major peptide fragment produced in the MS/MS spectra further also confirmed the characterization of peptides. As shown in Figure 1E, AR-9 could generate a variety of diagnostic product ions (DPIs) at *m/z* 72.08 (b_1_), *m/z* 171.11 (b_2_), *m/z* 318.19 (b_3_), *m/z* 175.11 (y_1_), *m/z* 232.13 (y_2_), *m/z* 331.21 (y_3_), *m/z* 444.29 (y_4_), *m/z* 531.33 (y_5_), *m/z* 628.38 (y_6_), and *m/z* 775.45 (y_7_). The molecular weight of the entire peptide was 944.54280. The fragment ion at *m/z* 72.08 was produced due to the loss of hydroxyl groups (17 Da) of alanine. The fragment ions at *m/z* 775.41 ([M-171.11+2H]^+^) and *m/z* 628.38 ([M-318.19+2H]^+^) indicated the presence of valine ([b_2_-b_1_+H_2_O]) and phenylalanine ([b_3_-b_2_+H_2_O]). Moreover, the loss of proline ([y_6_-y_5_+H_2_O]), serine ([y_5_-y_4_+H_2_O]), isoleucine ([y_4_-y_3_+H_2_O]), valine ([y_3_-y_2_+H_2_O]), glycine ([y_2_-y_1_+H_2_O]), and y_1_-arginine ([M + H]^+^) was detected. Therefore, the amino acid sequence of AR-9 was speculated as alanine–valine–phenylalanine–proline–serine–isoleucine–valine–glycine–arginine (AVFPSIVGR). In order to determine the correctness of the inferred structure, AR-9 was synthesized by solid-phase peptide synthesis and re-analyzed using UPLC-MS/MS with the same method. DPIs and retention time of synthetic were consistent with those of the isolated compound.

The physical and chemical indicators of AR-9 were predicted using the PepDraw database (http://pepdraw.com/) and the PEPFOLD3 database (https://bioserv.rpbs.univ-paris-diderot.fr/services/PEP-FOLD3/) (Figures 1F,G), and the results showed that the isoelectric point of 11.18 has good hydrophilicity for hydrophilicity index 8.21 kcal/mol. In addition, AR-9 should belong to a fragment for the actin protein of American cockroach (*Blatta americana*) using UniProt (https://www.uniprot.org/).

### AR-9 Ameliorated Lipid-Related Factors in Plasma and Lipid Accumulation in Hepatic Tissues in the HFD-Induced HL Model

**FIGURE 2:**
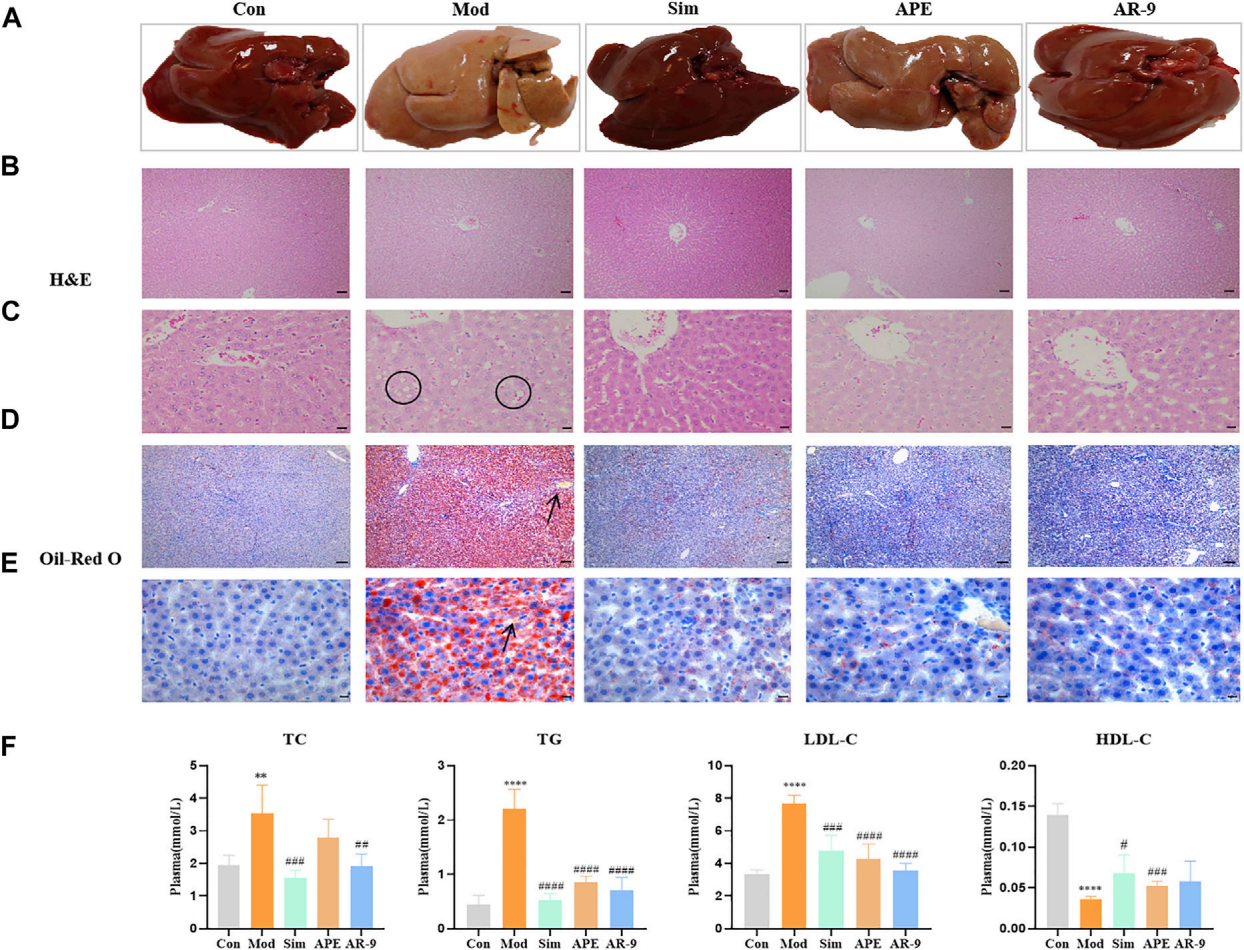
Results of anti-HL activity of AR-9. Con: control group, Mod: high-fat diet group, APE: APE treatment group, and Sim: simvastatin calcium administration group. **(A)** Rat liver photographs in test groups. **(B)** H&E staining (scale bar, 200 μm) of rat livers in groups. **(C)** H&E staining (scale bar, 50 μm) of rat livers in groups. **(D)** Oil Red O staining (scale bar, 500 μm) of rat livers in groups, and **(E)** Oil-Red O staining (scale bar, 50 μm) of rat livers in groups. **(F)** Hypolipidemic effect of AR-9 (TC, TG, LDL-C and HDL-C levels in the plasma of rats). n = 5–6. ^*^ *p* < 0.05, ^**^ *p* < 0.01, ^***^ *p* < 0.001, and ^****^ *p* < 0.0001 and ^#^ *p* < 0.05, ^##^ *p* < 0.01, ^###^ *p* < 0.001, and ^####^ *p* < 0.0001 vs. Con or Mod group. The arrows indicate lipid droplets. The circles indicate vacuolar hepatic steatosis.

To evaluate the lipid-lowering effect of AR-9, the rats were fed a HFD for 8 weeks to establish a HL model. Next, the rats were administered the corresponding drugs for 3 weeks. HFD feeding induced considerable abnormalities in blood lipids, as evidenced by the marked increase in the plasma levels of TC, TG, and LDL-C (*p* < 0.05) and the decrease of HDL-C compared with the levels in the normal diet–fed rats. After treatment, AR-9 and APE all mitigated these adverse changes, compared to the model group, and AR-9 and APE could reduce the levels of TG, TC, and LDL-C and increase the level of HDL-C. Interestingly, the ability of AR-9 to resist the lipid is stronger than that of APE (Figure 2F).

Simultaneously, we assessed the color, morphology, and lipid accumulation in the livers from different groups. As shown in Figures 2A–E, HFD feeding led to severe microvascular steatosis, hepatic steatosis, and increased lipid deposition. In contrast, AR-9 treatment significantly decreased lipid deposition and alleviated hepatic steatosis and liver injury compared with the observations in HL rats. A lipid-regulating effect was also observed in the Sim-treated rats.

### AR-9 Significantly Improved the Profile of Metabolite from Gut Microbiota in the HFD-Induced HL Model

**FIGURE 3:**
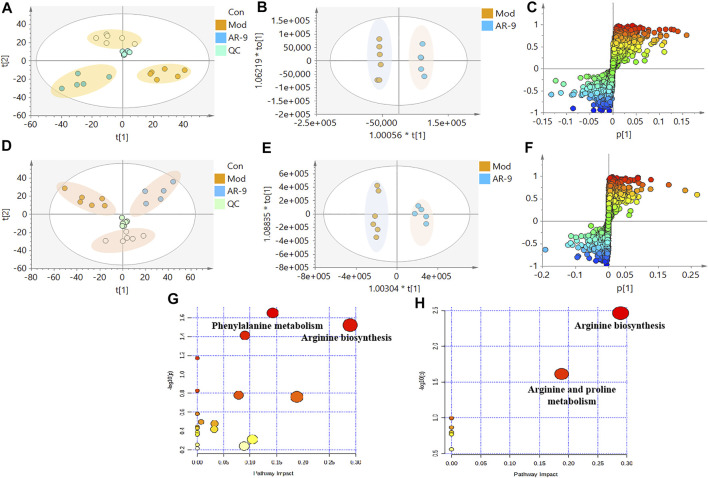
Effects of AR-9 on metabolic parameters in HL rats and correlation analysis. **(A)** Negative PCA score plot. **(D)** Positive PCA score plot. **(B)** Negative OPLS-DA plots (*R2X* = 0.315, *R2Y* = 0.997, *Q2* = 0.756, *p*-value of CV-ANOVA = 0.0473). **(E)** Positive OPLS-DA plots (*R2X* = 0.433, *R2Y* = 0.979, *Q2* = 0.795, *p*-value of CV-ANOVA = 0.0293). **(C)** Negative S-plots. **(F)** Positive S-plots. **(G)** Fecal sample pathway analysis between Con vs. Mod. **(H)** Fecal sample pathway analysis between AR-9 vs. Mod.

**FIGURE 4:**
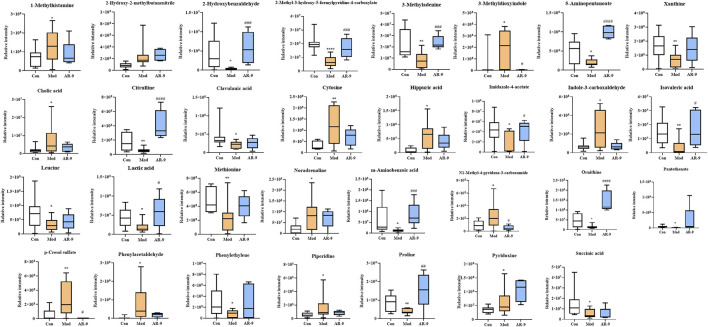
Results of 31 differential metabolites among three groups. ^*^ *p* < 0.05, ^**^ *p* < 0.01, ^***^ *p* < 0.001, and ^****^ *p* < 0.0001 and ^#^ *p* < 0.05, ^##^ *p* < 0.01, ^###^ *p* < 0.001, and ^####^ *p* < 0.0001 vs. Con or Mod group. AR-9 Treatment restored the dysbiosis of the gut microbiota in HL rats induced by HFD.

The data for the feces metabolic profiles of the Con, Mod, and AR-9 groups were patterned by an unsupervised PCA method. The results are shown in Figure 3A, D. Among them, QC samples were closely clustered, suggesting the stability of the system during sample analysis. Moreover, there was a clear separation between the three groups. For OPLS-DA analysis (Figures 3B,E), the feces metabolic profiles were significantly different between the Con group and the Mod group and the Mod group and AR-9 group. Likewise, seven-round cross-validation and 200 time-permutation testing showed that the OPLS-DA models were robust. Also, the S-plot scores (Figures 3C,F) could be used to screen and identify differential metabolites in the OPLS-DA model. Based on the threshold of VIP>1 and *p* < 0.05, finally, a total of 31 representative compounds showed significant changes between the Con and Mod groups. The results are shown in Table. 1; Figure 4. Among them, 11 metabolites were significantly increased in rats after being administered AR-9.

**TABLE 1:** Identified potential biomarkers regulated by AR-9.

Rt (min)	Theoretical *m/z*	Experimental *m/z*	Error (ppm)	Formula	Proposed identity	Change Trend (M/C)	Change Trend (AR-9/M)	Ion Mode
1.52	105.07001	105.06991	1.267	C_8_H_8_	Phenylethylene	↑^*^	↓	P
1.53	125.03456	125.03448	−0.591	C_5_H_6_N_2_O_2_	Imidazole-4-acetate	↓^*^	↑^#^	N
1.55	117.01819	117.01809	−1.240	C_4_H_6_O_4_	Succinic acid	↓^*^	-	N
1.81	187.00621	187.00623	1.466	C_7_H_8_O_4_S	p-Cresol sulfate	↑^**^	↓^#^	N
1.92	178.04991	178.05009	0.220	C_9_H_9_NO_3_	Hippuric acid	↑^*^	↓	N
2.03	220.11752	220.1181	0.685	C_9_H_17_NO_5_	Pantothenate	↓^*^	↑	P
2.04	180.02918	180.02948	0.346	C_8_H_7_NO_4_	2-Methyl-3-hydroxy-5-formylpyridine-4-carboxylate	↓^****^	↑^##^	N
2.23	89.02301	89.02306	−0.261	C_3_H_6_O_3_	Lactic acid	↓^*^	↑^#^	N
2.38	153.04033	153.04077	0.445	C_5_H_4_N_4_O_2_	Xanthine	↓^**^	↑	P
2.54	100.07585	100.07581	1.194	C_5_H_9_NO	2-hydroxy-2-methylbutanenitrile	↑^*^	↓	P
2.91	164.09261	164.0932	0.128	C_9_H_9_NO_2_	3-methyldioxyindole	↑^*^	↓^#^	P
3.03	170.08086	170.08118	0.060	C_8_H_11_NO_3_	Pyridoxine	↑^*^	↓	P
3.04	121.0647	121.06462	-0.756	C_8_H_8_O	Phenylacetaldehyde	↑^*^	↓	P
3.10	86.09687	86.09676	3.88	C_5_H_11_N	Piperidine	↑^*^	↓	P
3.48	153.06542	153.06581	−0.288	C_7_H_8_N_2_O_2_	N1-methyl-4-pyridone-3-carboxamide	↑^*^	↓^#^	P
3.92	101.0594	101.05955	−0.156	C_5_H_10_O_2_	Isovaleric acid	↓^**^	↑^#^	N
4.81	150.07709	150.05803	−1.972	C_5_H_11_NO_2_S	Methionine	↓^**^	↑	P
4.84	148.06170	148.06186	0.088	C_6_H_7_N_5_	3-methyladenine	↓^**^	↑^###^	N
4.85	112.05053	112.05055	0.104	C_4_H_5_N_3_O	Cytosine	↑^**^	↓	P
4.90	170.08073	170.08121	0.237	C_8_H_11_NO_3_	Noradrenaline	↑^*^	↓	P
8.22	198.03997	198.04018	2.430	C_8_H_9_NO_5_	Clavulanic acid	↓^*^	↑	N
9.01	146.05984	146.05972	−2.194	C_9_H_7_NO	Indole-3-carboxaldehyde	↑^*^	↓	P
9.02	126.10226	126.10238	−1.538	C_6_H_11_N_3_	1-methylhistamine	↑^*^	↓	P
9.21	132.10044	132.10164	−2.008	C_6_H_13_NO_2_	Leucine	↓^*^	↑	P
9.36	118.08625	118.08623	−0.044	C_5_H_11_NO_2_	5-aminopentanoate	↓^*^	↑^####^	P
9.38	121.06453	121.02837	−0.297	C_7_H_5_O_2_	2-hydroxybenzaldehyde	↓^*^	↑^###^	N
9.80	116.07063	116.07059	0.215	C_5_H_9_NO_2_	Proline	↓^**^	↑^#^	P
10.20	138.05469	138.05473	−1.630	C_7_H_7_NO_2_	m-Aminobenzoic acid	↓^*^	↑^###^	P
10.28	407.28003	407.28003	2.036	C_24_H_40_O_5_	Cholic acid	↑^*^	↓	N
10.30	176.10257	176.10292	−0.272	C_6_H_13_N_3_O_3_	Citrulline	↓^**^	↑^####^	P
10.34	131.08132	131.0815	−0.004	C_5_H_12_N_2_O_2_	Ornithine	↓^*^	↑^####^	N

^*^ *p* < 0.05, ^**^ *p* < 0.01, ^***^ *p* < 0.001, and ^****^ *p* < 0.0001 and ^#^ *p* < 0.05, ^##^ *p* < 0.01, ^###^ *p* < 0.001, and ^####^ *p* < 0.0001 vs. control and model group.

To clarify the exact pathways of AR-9 resisting HL, differential metabolites with Con vs. Mod were imported into the MetaboAnalyst database (MetaboAnalyst). The results of the pathway analysis revealed that there were three primary disturbed pathways (*p* < 0.05) of feces between Con vs*.* Mod, involving 1) phenylalanine metabolism; 2) arginine biosynthesis; 3) histidine metabolism. Moreover, there were two primary disturbed pathways (*p* < 0.05) of feces between AR-9 vs. Mod, involving 1) arginine biosynthesis and 2) arginine and proline metabolism. The color and size of each circle in Figures 3G,H were based on the *p*-values and pathway impact values, respectively. The results suggested that the disturbed pathways in response to HL and AR-9 treatment were mainly arginine biosynthesis.

In this study, 18 fecal samples from the Con, Mod, and AR-9 groups were used to evaluate the ameliorating effect of AR-9 on the symptoms of HL in rats. The high-throughput sequencing yielded a total of 1,463,160 reads, which passed all quality filters with a 97% identity threshold to obtain a total of 11,017 species classification OTUs.

**FIGURE 5:**
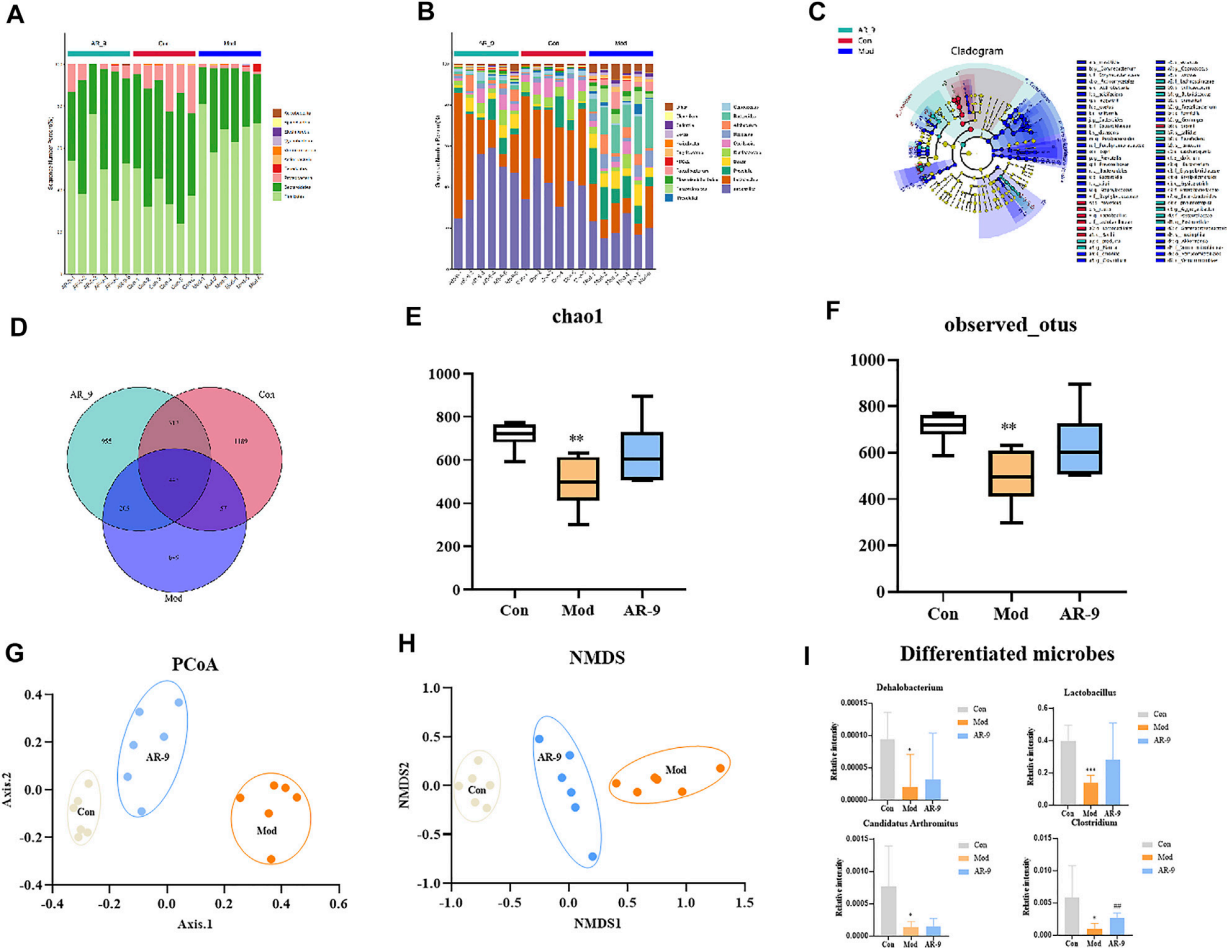
Intestinal microbiota analysis results of AR-9 treatment of HL. The histogram of species distribution at the phylum **(A)** and genus **(B)** levels in three groups was revealed by 16S rRNA sequencing (different colors represent different bacteria at phylum or genus levels). **(C)** Taxonomic cladogram generated by LEfSe analysis showing the differences of bacterial abundance on the genus level. **(D)** Venn diagram for bacterial communities of Con, Mod, and AR-9 groups. **(E)** Chao1 and **(F)** observed OTUs represent alpha-diversity indexes for each sample group. **(G)** PCoA analysis among Con, Mod, and AR-9 groups. **(H)** NMDS analysis among Con, Mod and AR-9 groups. **(I)** Results of four differential dominant bacteria at the genus level among AR-9 groups. ^*^ *p* < 0.05, ^**^ *p* < 0.01 and ^#^ *p* < 0.05, and ^##^ *p* < 0.01 vs. Con or Mod group.

To verify whether the sequencing amount of this study was large enough to reflect the diversity of the original microorganisms, Alpha diversity analyses (e. g. Chao1, observed OTUs, Faith’s Phylogenetic Distance, Shannon, and Simpson) were conducted on the results of 16S rRNA sequencing based on the 97% similarity level. It could be found from Figures 5E,F that the Con group presented a more diverse bacterial community than the Mod group. Furthermore, bacterial diversity was also increased in rats treated with AR-9. Venn plots could visually show the structural similarity and overlap of species in different groups of samples (Wang et al., 2016). Overall, the Mod, Con, and AR-9 groups shared 445 common species, while the AR-9 group had 955 unique species and the Mod group had 649 unique species (Figure 5D).

The beta diversities were described by a principal component analysis (PCoA analysis) and a non-metric multidimensional scaling (NMDS) analysis. Both PCoA (Figure 5G) and NMDS (Figure 5H) analyses showed that there were different clusters among the three groups, suggesting significant community differences. A long-term HFD led to a decrease in the number of OTUs in rats, but the situation reversed with AR-9 intervention. Taken together, the results demonstrated that AR-9 treatment modulated gut flora dysbiosis in HFD-induced rats, resulting in the restoration of a microbial community similar to that in controls.

Figures 5A,B show the results of species annotation analysis at the phylum and genus levels in three groups. At the phylum level, *Firmicutes* and *Bacteroidetes* were the most predominant phylum in gut bacteria of rats and account for the mainly relative abundance in all samples, as shown in Figure 5A. Compared with the Con group, *Bacteroidetes* in the Mod group decreased, while *Firmicutes* increased. In contrast, compared to the Mod group, *Firmicutes* decreased in AR-9 group, whereas *Bacteroidetes* increased. The differently abundant taxa among experimental samples were identified using linear discriminant analysis effect size (LEfSe) (Ge et al., 2021). The analysis was performed at the taxonomic level from phylum to genus (Figure 5C). In terms of the whole genera composition, a total of 12 genera showed significant differences in the Con group compared with those in the Mod group, of which four taxa were more abundant in AR-9 than that in Mod, while only one taxon was significantly more abundant (*p* < 0.05). The relative abundance of selected taxa in the Mod group relative to the AR-9 group is shown in Figure 5I. According to the results, the four bacterial genera with significant differences in relative abundance were *Lactobacillus*, *Clostridium*, *Dehalobacterium*, and *Candidatus Arthromitus.* Especially, *Clostridium* was present in a significantly lower proportion in the Mod group than the AR-9 group (*p* < 0.01).

### Spearman’s Correlation Analysis Revealed the Mechanism of AR-9 Against HL.

**FIGURE 6:**
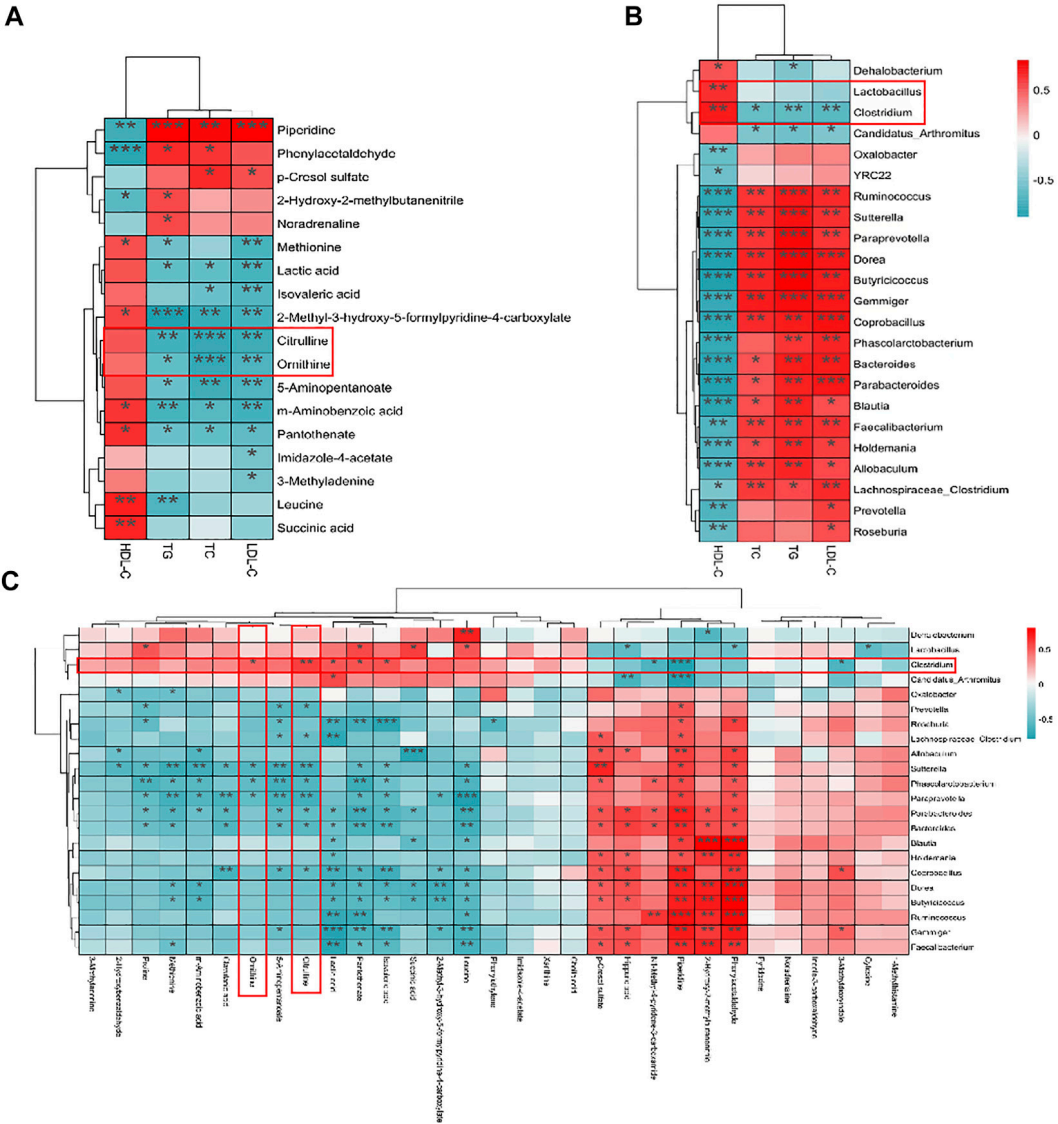
Spearman’s correlation analysis results of omics data. **(A)** Heatmap of the correlations between plasma lipid parameters and fecal metabolites. **(B)** Heatmap of the correlations between plasma lipid parameters and microbial genus abundances. **(C)** Heatmap of the correlations between microbial genus abundances and fecal metabolites. ^*^ *p* < 0.05, ^**^ *p* < 0.01, and ^***^ *p* < 0.001 vs. Con group.

The correlations, which were applied to represent the covariation in perturbed gut flora genus, altered fecal metabolites, and lipid factors were analyzed by the Spearman correlation analysis. As shown in Figure 6A, the result between lipid factors and altered fecal metabolites revealed that multiple metabolites, such as citrulline, ornithine, lactic acid, m-aminobenzoic acid, 5-aminopentanoate, pantothenate, and 2-methyl-3-hydroxy-5-formylpyridine-4-carboxylate, showed significantly negative (*p* < 0.05) correlations with TC, TG, and LDL-C levels. These results indicated that the abovementioned compounds might be pivotal metabolites of the beneficial function of AR-9. Hereafter, the correlations between gut flora and lipid factors were revealed (Figure 6B). Among them, the relative abundances of *Clostridium* and *Candidatus Arthromitus* showed negative correlations with TC, TG, and LDL-C levels, while only the relative abundance of *Clostridium* exhibited a positive correlation of HDL-C. Simultaneously, the relative abundances of 14 bacteria showed significantly positive trends with TC, TG, and LDL-C and the obviously negative correlations of HDL-C. Certainly, the close correlations between gut flora and metabolites were also found (Figure 6C), and the correlation results showed that *Clostridium* had significant positive correlations between citrulline and ornithine. The abovementioned results indicated that AR-9 mostly regulated the arginine biosynthesis pathway *via* acting on *Clostridium*. Moreover, citrulline and ornithine should be used as biomarkers of AR-9 in the treatment of HL.

## Discussion

The first-line medicine for the treatment of HL includes three categories: statins, fibrates, and niacin (Oh et al., 2020). The abovementioned drugs have clear curative effects on HL, but their obvious side effects are also extremely prominent. Bioactive peptides, which are especially obtained from plants and animals, have been reported to exhibit the hypolipidemic activity accompanied by their securities (Shen et al., 2017). Previously, most peptide drugs were injected into the circulatory system to prevent and treat diseases. Oral delivery of peptide therapeutics as a convenient alternative to injections has been an area of research for the pharmaceutical scientific community for the last several decades (Tyagi et al., 2018). In this study, AR-9 was separated from APE with ion-exchange chromatography, and its structure was also characterized by multiple analysis methods. Meanwhile, the HFD-induced HL SD rat model was established to evaluate the anti-HL activity of AR-9. The results showed that AR-9 could not only significantly reduce TG, TC, and LDL levels (*p* < 0.05) but also increase HDL amounts in plasma. In addition, AR-9 reduced the accumulation of lipids in the liver based on Oil Red O staining and alleviated liver damage, which was caused by HFD.

In order to find out the mechanism of AR-9 against HL, the profiles of gut flora and its metabolites were studied in detail by 16S rRNA sequencing and fecal metabolomics. In gut flora results, a HFD significantly increased the relative abundance of *Firmicutes* and decreased that of *Bacteroidetes*; however, AR-9 reversed this trend. At the genus level, AR-9 could upregulate the relative abundance of four bacteria: *Lactobacillus*, *Clostridium*, *Dehalobacterium*, and *Candidatus Arthromitus*. Among them, *Lactobacillus* and *Clostridium* were directly related to metabolic diseases, such as type 2 diabetes, HL, atherosclerosis, and hepatic steatosis (Cotillard et al., 2013; Staley et al., 2017). The connection was mainly embodied through metabolites produced by the two bacterial groups, including short-chain fatty acids, amino acids, and their derivatives.

Many studies had shown a positive correlation between HL and amino acid metabolism, such as tryptophan metabolism and arginine and proline metabolism. (Liang et al., 2021). In the present study, an analysis strategy of metabolic profiling of fecal endogenous metabolites was utilized to further explore the beneficial effect and the potential mechanism of AR-9 in relieving HL. The results showed that biomarkers of AR-9 against HL were ornithine and citrulline. Citrulline is a substrate for renal arginine synthesis, and arginine is the precursor of NO, which is the most important vasodilator generated by vascular endothelial cells under NO synthase (Li et al., 2016). The NO supplementation can activate AMP-activated protein kinase (AMPK) and its downstream targets to inhibit *de novo* lipid biosynthesis (Khalaf et al., 2019). Meanwhile, ornithine, as another degradation product of arginine, plays an important role in the treatment of metabolic diseases. This function may be related to the ability of ornithine to stimulate glucose-dependent insulin secretion and inhibit the rate of sugar-to-lipid conversion.

**FIGURE 7:**
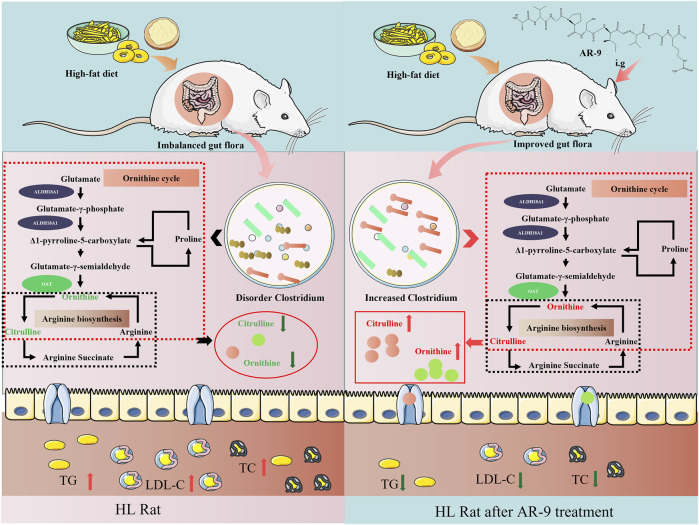
Effects of AR-9 in the rat with HL. The green arrows indicate downregulation effects, and the red arrows represent upregulation effects.

Citrulline and ornithine are produced under the fermentation of gut flora, proteins, and bioactive peptides. *Lactobacillus*, *Bacteroides*, and *Clostridium* have been illustrated to have the abovementioned function (Staley et al., 2017). Coincidentally, in this study, the results of Spearman’s correlation analysis between perturbed gut flora genus, altered fecal metabolites, and lipid factors showed that AR-9 may directly upregulate the levels of citrulline and ornithine *via* the metabolic function of *Clostridium* to achieve a beneficial effect of treating HL (Figure 7). However, the truly interactive relationship between AR-9 and *Clostridium* has not been revealed and verified. Many studies have confirmed that active peptides can be more easily absorbed into the circulatory system by converting to small molecules (Shen et al., 2017). Moreover, the activities of the original peptides may also be manifested by pathways mediated by the small molecules. Future studies will be needed to verify the proposed mechanism by which AR-9 exerts anti-hyperlipidemia effects in the present study.

## Conclusion

In this study, the effects of AR-9 on lipid factors and gut flora in SD rats with HFD-induced HL were studied. AR-9 could regulate the levels of TG, TC, LDL-C, and HDL-C in plasma and repair the damaged liver function. For gut flora and metabolomics, AR-9 mostly regulated the arginine biosynthesis pathway *via* acting on *Clostridium*.

Dyslipidemia, the disorder of lipid metabolism, and decreased diversity and abundance of gut flora were ubiquitous in HL. It was proven by metabolomics that the metabolic profile of the feces of the rats in the HL group changed significantly. At the same time, the effect of AR-9 on preventing HL was evaluated. The existence of a link between metabolic biomarkers and the gut microbial has an important influence on the host. AR-9 can regulate amino acid metabolism, increase the diversity and abundance of gut flora, regulate the structure of gut flora, and thus contribute to health. This study identified key functional bacteria and potential biomarkers of HL in rats and established a correlation between them and AR-9 treatment. All results in this study will provide support for the continued development of AR-9.

There are some limitations of the present study. First, the identified differences in gut microbials and metabolites for diagnostic and prognostic evaluations were not verified by further study. Second, AR-9 is administered in a single dose. Third, the interactive relationships between AR-9 and *Clostridium* and anti-HL of AR-9 and its absorbed components urgently should be resolved. Accordingly, the findings of the current study will be further investigated for uncovering the metabolic and microbial basis of AR-9 in the treatment of HL.

## Data Availability

The original contributions presented in the study are publicly available. This data can be found in Sequence Read Archive (https://www.ncbi.nlm.nih.gov/sra) using the accession number PRJNA566284.

## References

[B1] Alanee S. El-Zawahry A. Dynda D. Dabaja A. McVary K. Karr M. (2019). A Prospective Study to Examine the Association of the Urinary and Fecal Microbiota with Prostate Cancer Diagnosis after Transrectal Biopsy of the Prostate Using 16sRNA Gene Analysis. Prostate 79, 81–87. 10.1002/pros.23713 30117171

[B2] Cotillard A. Kennedy S. P. Kong L. C. Prifti E. Pons N. Le Chatelier E. (2013). Dietary Intervention Impact on Gut Microbial Gene Richness. Nature 500, 585–588. 10.1038/nature12480 23985875

[B3] Ge H. Cai Z. Chai J. Liu J. Liu B. Yu Y. (2021). Egg White Peptides Ameliorate Dextran Sulfate Sodium-Induced Acute Colitis Symptoms by Inhibiting the Production of Pro-inflammatory Cytokines and Modulation of Gut Microbiota Composition. Food Chem. 360, 129981. 10.1016/j.foodchem.2021.129981 34020366

[B4] Guthals A. Bandeira N. (2012). Peptide Identification by Tandem Mass Spectrometry with Alternate Fragmentation Modes. Mol. Cell Proteomics 11, 550–557. 10.1074/mcp.R112.018556 22595789 PMC3434779

[B5] Hackshaw K. V. Miller J. S. Aykas D. P. Rodriguez-Saona L. (2020). Vibrational Spectroscopy for Identification of Metabolites in Biologic Samples. Molecules 25, 4725. 10.3390/molecules25204725 PMC7587585 33076318

[B6] Hall M. Beiko R. G. Clifton N. J. (2018). 16S rRNA Gene Analysis with QIIME2. Methods Mol. Biol. 1849, 113–129. 10.1007/978-1-4939-8728-3_8 30298251

[B7] He J. Unser S. Bruzas I. Cary R. Shi Z. Mehra R. (2018). The Facile Removal of CTAB from the Surface of Gold Nanorods. Colloids Surf. B Biointerfaces 163, 140–145. 10.1016/j.colsurfb.2017.12.019 29291499

[B8] Huang F. Zheng X. Ma X. Jiang R. Zhou W. Zhou S. (2019). Theabrownin from Pu-Erh Tea Attenuates Hypercholesterolemia via Modulation of Gut Microbiota and Bile Acid Metabolism. Nat. Commun. 10, 4971. 10.1038/s41467-019-12896-x 31672964 PMC6823360

[B9] Indiani C. M. D. S. P. Rizzardi K. F. Castelo P. M. Ferraz L. F. C. Darrieux M. Parisotto T. M. (2018). Childhood Obesity and Firmicutes/Bacteroidetes Ratio in the Gut Microbiota: A Systematic Review. Child. Obes. 14, 501–509. 10.1089/chi.2018.0040 30183336

[B10] Khalaf D. Krüger M. Wehland M. Infanger M. Grimm D. (2019). The Effects of Oral L-Arginine and L-Citrulline Supplementation on Blood Pressure. Nutrients 11, 1679. 10.3390/nu11071679 PMC6683098 31336573

[B11] Li B. He X. Jia W. Li H. (2017). Novel Applications of Metabolomics in Personalized Medicine: A Mini-Review. Molecules 22, 1173. 10.3390/molecules22071173 PMC6152045 28703775

[B12] Li Q. Gu W. Ma X. Liu Y. Jiang L. Feng R. (2016). Amino Acid and Biogenic Amine Profile Deviations in an Oral Glucose Tolerance Test: A Comparison between Healthy and Hyperlipidaemia Individuals Based on Targeted Metabolomics. Nutrients 8, 379. 10.3390/nu8060379 PMC4924220 27338465

[B13] Li W. Jiang N. Li B. Wan M. Chang X. Liu H. (2018). Antioxidant Activity of Purified Ulvan in Hyperlipidemic Mice. Int. J. Biol. Macromol. 113, 971–975. 10.1016/j.ijbiomac.2018.02.104 29476858

[B14] Liang J. Kou S. Chen C. Raza S. H. A. Wang S. Ma X. (2021). Effects of Clostridium Butyricum on Growth Performance, Metabonomics and Intestinal Microbial Differences of Weaned Piglets. BMC Microbiol. 21, 85. 10.1186/s12866-021-02143-z 33752593 PMC7983215

[B15] Liu H. Yan Y. Zhang F. Wu Q. (2019). The Immuno-Enhancement Effects of Tubiechong *(Eupolyphaga Sinensis)* Lyophilized Powder in Cyclophosphamide-Induced Immunosuppressed Mice. Immunol. Invest. 48, 844–859. 10.1080/08820139.2019.1588291 30917711

[B16] Liu L. L. Wang L. Zonderman J. Rouse J. C. Kim H. Y. (2020). Automated, High-Throughput Infrared Spectroscopy for Secondary Structure Analysis of Protein Biopharmaceuticals. J. Pharm. Sci. 109, 3223–3230. 10.1016/j.xphs.2020.07.030 32758548

[B17] Lopes J. L. Miles A. J. Whitmore L. Wallace B. A. (2014). Distinct Circular Dichroism Spectroscopic Signatures of Polyproline II and Unordered Secondary Structures: Applications in Secondary Structure Analyses. Protein Sci. 23, 1765–1772. 10.1002/pro.2558 25262612 PMC4253816

[B18] Love M. I. Huber W. Anders S. (2014). Moderated Estimation of Fold Change and Dispersion for RNA-Seq Data with DESeq2. Genome Biol. 15, 550. 10.1186/s13059-014-0550-8 25516281 PMC4302049

[B19] Mandal S. Van Treuren W. White R. A. Eggesbø M. Knight R. Peddada S. D. (2015). Analysis of Composition of Microbiomes: a Novel Method for Studying Microbial Composition. Microb. Ecol. Health Dis. 26, 27663. 10.3402/mehd.v26.27663 26028277 PMC4450248

[B20] Mant C. T. Hodges R. S. (2016). Separation of Peptides on HALO 2-Micron Particles. Curr. Protoc. Protein Sci. 85, 11–16. 10.1002/cpps.12 27479502

[B21] Nicholson J. K. Holmes E. Kinross J. Burcelin R. Gibson G. Jia W. (2012). Host-gut Microbiota Metabolic Interactions. Science 336, 1262–1267. 10.1126/science.1223813 22674330

[B22] Oh R. C. Trivette E. T. Westerfield K. L. (2020). Management of Hypertriglyceridemia: Common Questions and Answers. Am. Fam. Physician 102, 347–354. 32931217

[B23] Owens A. P. 3rd Byrnes J. R. Mackman N. (2014). Hyperlipidemia, Tissue Factor, Coagulation, and Simvastatin. Trends Cardiovasc Med. 24, 95–98. 10.1016/j.tcm.2013.07.003 24016468 PMC4102256

[B24] Rom O. Liu Y. Liu Z. Zhao Y. Wu J. Ghrayeb A. (2020). Glycine-based Treatment Ameliorates NAFLD by Modulating Fatty Acid Oxidation, Glutathione Synthesis, and the Gut Microbiome. Sci. Transl. Med. 12, eaaz2841. 10.1126/scitranslmed.aaz2841 33268508 PMC7982985

[B25] Shen W. Matsui T. (2017). Current Knowledge of Intestinal Absorption of Bioactive Peptides. Food Funct. 8, 4306–4314. 10.1039/c7fo01185g 29139513

[B26] Singh A. Sharma D. Varghese L. M. Mahajan R. (2020). Fast Flow Rate Processes for Purification of Alkaline Xylanase Isoforms from Bacillus Pumilus AJK and Their Biochemical Characterization for Industrial Application Purposes. Biotechnol. Prog. 36, e2898. 10.1002/btpr.2898 31469503

[B27] Staley C. Weingarden A. R. Khoruts A. Sadowsky M. J. (2017). Interaction of Gut Microbiota with Bile Acid Metabolism and its Influence on Disease States. Appl. Microbiol. Biotechnol. 101, 47–64. 10.1007/s00253-016-8006-6 27888332 PMC5203956

[B28] Tatulian S. A. (2019). FTIR Analysis of Proteins and Protein-Membrane Interactions. Methods Mol. Biol. Clift. 2003, 281–325. 10.1007/978-1-4939-9512-7_13 31218623

[B29] Tiernan H. Byrne B. Kazarian S. G. (2020). ATR-FTIR Spectroscopy and Spectroscopic Imaging for the Analysis of Biopharmaceuticals. Spectrochim. Acta A Mol. Biomol. Spectrosc. 241, 118636. 10.1016/j.saa.2020.118636 32610215 PMC7308041

[B30] Tyagi P. Pechenov S. Anand Subramony J. (2018). Oral Peptide Delivery: Translational Challenges Due to Physiological Effects. J. Control Release 287, 167–176. 10.1016/j.jconrel.2018.08.032 30145135

[B31] Wang Y. Xu L. Gu Y. Q. Coleman-Derr D. (2016). MetaCoMET: a Web Platform for Discovery and Visualization of the Core Microbiome. Bioinformatics 32, 3469–3470. 10.1093/bioinformatics/btw507 27485442

[B32] Yang Y. Misra B. B. Liang L. Bi D. Weng W. Wu W. (2019). Integrated Microbiome and Metabolome Analysis Reveals a Novel Interplay between Commensal Bacteria and Metabolites in Colorectal Cancer. Theranostics 9, 4101–4114. 10.7150/thno.35186 31281534 PMC6592169

[B33] Yao Y. S. Li T. D. Zeng Z. H. (2020). Mechanisms Underlying Direct Actions of Hyperlipidemia on Myocardium: an Updated Review. Lipids Health Dis. 19, 23. 10.1186/s12944-019-1171-8 32035485 PMC7007679

[B34] Yu M. Jia H. Zhou C. Yang Y. Zhao Y. Yang M. (2017). Variations in Gut Microbiota and Fecal Metabolic Phenotype Associated with Depression by 16S rRNA Gene Sequencing and LC/MS-based Metabolomics. J. Pharm. Biomed. Anal. 138, 231–239. 10.1016/j.jpba.2017.02.008 28219800

[B35] Zhang N. Zhao Y. Shi Y. Chen R. Fu X. Zhao Y. (2019). Polypeptides Extracted from Eupolyphaga Sinensis Walker *via* Enzymic Digestion Alleviate UV Radiation-Induced Skin Photoaging. Biomed. Pharmacother. 112, 108636. 10.1016/j.biopha.2019.108636 30802824

[B36] Zhang Q. Wu Y. Wang J. Wu G. Long W. Xue Z. (2016). Accelerated Dysbiosis of Gut Microbiota during Aggravation of DSS-Induced Colitis by a Butyrate-Producing Bacterium. Sci. Rep. 6, 27572. 10.1038/srep27572 27264309 PMC4893749

[B37] Zheng Z. Y. Cao F. W. Wang W. J. Yu J. Chen C. Chen B. (2020). Probiotic Characteristics of Lactobacillus Plantarum E680 and its Effect on Hypercholesterolemic Mice. BMC Microbiol. 20, 239. 10.1186/s12866-020-01922-4 32753060 PMC7401229

